# Tuberculosis decline in populations affected by HIV: a retrospective study of 12 countries in the WHO African Region

**DOI:** 10.2471/BLT.18.228577

**Published:** 2019-04-30

**Authors:** Christopher Dye, Brian G Williams

**Affiliations:** a Department of Zoology and All Souls College, University of Oxford, High Street, Oxford OX1 4AL, England.; b South African Centre for Epidemiological Modelling and Analysis (SACEMA), Stellenbosch University, Stellenbosch, South Africa.

## Abstract

**Objective:**

To investigate which of the World Health Organization recommended methods for tuberculosis control have had the greatest effect on case incidence in 12 countries in the World Health Organization (WHO) African Region that carry high burdens of tuberculosis linked to human immunodeficiency virus (HIV) infection.

**Methods:**

We obtained epidemiological surveillance, survey and treatment data on HIV and tuberculosis for the years 2003 to 2016. We used statistical models to examine the effects of antiretroviral therapy (ART) and isoniazid preventive therapy in reducing the incidence of tuberculosis among people living with HIV. We also investigated the role of tuberculosis case detection and treatment in preventing *Mycobacterium tuberculosis* transmission and consequently reducing tuberculosis incidence.

**Findings:**

Between 2003 and 2016, ART provision was associated with the decline of tuberculosis in each country, and with differences in tuberculosis decline between countries. Inferring that ART was a cause of tuberculosis decline, ART prevented 1.88 million (95% confidence interval, CI: 1.65 to 2.11) tuberculosis cases in people living with HIV, or 15.7% (95% CI: 13.8 to 17.6) of the 11.96 million HIV-positive tuberculosis cases expected. Population coverage of isoniazid preventive therapy was too low (average 1.0% of persons eligible) to have a major effect on tuberculosis decline, and improvements in tuberculosis detection and treatment were either weakly associated or not significantly associated with tuberculosis decline.

**Conclusion:**

ART provision is associated with tuberculosis decline in these 12 countries. ART should remain central to tuberculosis control where rates of tuberculosis–HIV coinfection are high, but renewed efforts to treat tuberculosis are needed.

## Résumé

**Objectif:**

Rechercher quelle méthode recommandée par l'Organisation mondiale de la Santé pour lutter contre la tuberculose a eu le plus gros effet sur l'incidence de la maladie dans 12 pays de la Région africaine de l’Organisation mondiale de la Santé (OMS) fortement touchés par la tuberculose en lien avec le virus de l'immunodéficience humaine (VIH).

**Méthodes:**

Nous nous sommes procuré des données épidémiologiques de surveillance, d'enquête et de traitement concernant le VIH et la tuberculose pour les années 2003 à 2016. Nous avons utilisé des modèles statistiques pour examiner les effets des traitements antirétroviraux (TAR) et des traitements préventifs par isoniazide sur la réduction de l'incidence de la tuberculose chez les personnes atteintes du VIH. Nous avons également analysé le rôle que jouent le dépistage et le traitement de la tuberculose dans la prévention de la transmission de *Mycobacterium tuberculosis* et donc dans la réduction de l'incidence de la maladie.

**Résultats:**

Entre 2003 et 2016, la prise de TAR a été associée à un déclin de la tuberculose dans tous les pays, avec des différences dans le déclin de la maladie selon les pays. En déduisant que les TAR étaient une des raisons du déclin de la tuberculose, ceux-ci ont empêché 1,88 million (intervalle de confiance de 95%, IC: 1,65 à 2,11) de cas de tuberculose chez les personnes atteintes du VIH, soit 15,7% (IC 95%: 13,8 à 17,6) des 11,96 millions de cas de tuberculose attendus chez des patients séropositifs au VIH. La couverture des traitements préventifs par isoniazide dans la population était trop faible (en moyenne 1,0% des personnes éligibles) pour avoir un effet notable sur le déclin de la tuberculose, et les améliorations apportées à la détection et au traitement de la maladie étaient associées faiblement ou de manière non significative au déclin de la tuberculose.

**Conclusion:**

Les TAR sont associés au déclin de la tuberculose dans ces 12 pays. Les TAR devraient rester au cœur de la lutte contre la tuberculose lorsque le taux de co-infection tuberculose/VIH est élevé, mais un regain d'efforts est également nécessaire pour combattre la tuberculose.

## Resumen

**Objetivo:**

Investigar cuáles de los métodos recomendados por la Organización Mundial de la Salud para el control de la tuberculosis han tenido el mayor efecto en la incidencia de casos en 12 países de la región africana de la Organización Mundial de la Salud (OMS) que soportan altas cargas de tuberculosis relacionadas con la infección por el virus de la inmunodeficiencia humana (VIH).

**Métodos:**

Se obtuvieron datos de vigilancia epidemiológica, encuestas y tratamientos sobre el VIH y la tuberculosis para los años 2003 a 2016. Se utilizaron modelos estadísticos para examinar los efectos de la terapia antirretroviral (TAR) y la terapia preventiva con isoniazida en la reducción de la incidencia de la tuberculosis entre las personas que viven con el VIH. También se investigó el papel de la detección y el tratamiento de casos de tuberculosis en la prevención de la transmisión de *Mycobacterium tuberculosis* y, en consecuencia, en la reducción de la incidencia de la tuberculosis.

**Resultados:**

Entre 2003 y 2016, la provisión de TAR se asoció con la disminución de la tuberculosis en todos los países y con diferencias en el descenso de la tuberculosis entre los países. Deduciendo que la TAR era una causa del descenso de la tuberculosis, la TAR previno 1,88 millones (intervalo de confianza, IC del 95 %: 1,65 a 2,11) de casos de tuberculosis en personas que viven con el VIH, o el 15,7 % (IC del 95 %: 13,8 a 17,6) de los 11,96 millones de casos de tuberculosis seropositiva previstos. La cobertura de la población del tratamiento preventivo con isoniazida era demasiado baja (una media del 1,0 % de las personas admisibles) como para tener un efecto importante sobre el descenso de la tuberculosis y las mejoras en la detección y el tratamiento de la tuberculosis se asociaron débilmente o no se asociaron de forma significativa con el descenso de la tuberculosis.

**Conclusión:**

La provisión de la terapia antirretroviral está asociada con el descenso de la tuberculosis en estos 12 países. La TAR debe seguir siendo fundamental para el control de la tuberculosis, donde las tasas de coinfección por tuberculosis y VIH son elevadas, pero es necesario redoblar los esfuerzos para tratar la tuberculosis.

## ملخص

**الغرض:**

اكتشاف أي من الطرق التي أوصت بها منظمة الصحة العالمية لمكافحة السل، كان لديها التأثير الأكبر على حدوث الإصابة في 12 بلداً إفريقياً في المنطقة الأفريقية لمنظمة الصحة العالمية(WHO) من تلك التي تتحمل أعباءً عالية من مرض السل مرتبطة بالإصابة بفيروس نقص المناعي البشرية ** (HIV) **لدى الإنسان **. **

**الطريقة:**

لقد حصلنا على بيانات عن مراقبة الأمراض الوبائية، وبيانات المسح والعلاج الخاصة بفيروس فيروس نقص المناعة البشرية والسل خلال الأعوام من 2003 إلى 2016. كما قمنا بالاستعانة بنماذج إحصائية لدراسة آثار العلاج بمضادات الفيروسات القهقرية ( **ART **) وعلاج أيزونيازيد الوقائي للحد من حدوث الإصابة بالسل بين الأشخاص المصابين بفيروس نقص المناعة البشرية. قمنا كذلك بالبحث في دور الكشف عن حالات السل وعلاجها في منع انتقال السل.

**النتائج:**

بين عامي 2003 و2016، ارتبط توفير العلاج بمضادات الفيروسات القهقرية بانخفاض في مرض السل في كل بلد، مع وجود اختلافات في انخفاض الإصابة بمرض السل بين البلدان. وباستنتاج أن مضادات الفيروسات القهقرية قد أدت إلى انخفاض معدل الإصابة بمرض السل، فقد أدت هذه المضادات لوقاية 1.88 مليون (فاصل الثقة 95%: من 1.65 إلى 2.11) حالة من الإصابة بمرض السل بين الأشخاص بفيروس نقص المناعة البشرية، أو 15.7% (فاصل ثقة 95%: 13.8 إلى 17.6) من 11.96 مليون حالة للإصابة المتوقعة بمرض السل لدى الأشخاص الذين يعانون من فيروس نقص المناعة البشرية. كانت التغطية السكانية لعلاج أيزونيازيد الوقائي منخفضة للغاية (بمتوسط 1.0٪ من الأشخاص المؤهلين) ليكون لها تأثير كبير على انخفاض الإصابة بمرض السل، وكانت التحسينات في اكتشاف مرض السل وعلاجه إما مرتبطة بشكل ضعيف أو غير مرتبطة بشكل ملموس بانخفاض الإصابة بمرض السل.

**الاستنتاج:**

ارتبط توفير العلاج بمضادات الفيروسات القهقرية بانخفاض في مرض السل في هذه البلدان الإثنا عشر. يجب أن يظل العلاج بمضادات الفيروسات القهقرية كحجر للزاوية في مكافحة السل حيث تكون معدلات الإصابة بمرض السل وفيروس نقص المناعة البشرية معاً مرتفعة، إلا أن هناك حاجة لجهود متجددة لعلاج مرض وعدوى السل **. **

## 摘要

**目的:**

旨在研究哪些世界卫生组织推荐的结核病防控方法对世卫组织非洲区域 12 国的发病率影响最大,这些国家与人体免疫缺陷病毒 (HIV) 感染有关的肺结核疾病负担沉重。

**方法:**

我们获得了 2003 年至 2016 年间的艾滋病和结核病的流行病学监测、调查和治疗数据。我们采用统计模型来检测抗逆转录病毒疗法 (ART) 和异烟肼预防性疗法在降低艾滋病毒感染者中结核病发病率方面的疗效。我们还研究了结核病病例检测和治疗在预防*结核分枝杆菌*传播和减少结核病发病率方面的作用。

**结果:**

在 2003 年至 2016 年间,抗逆转录病毒疗法 (ART) 与各国结核病发病率的降低和不同国家间下降率的差异均有关联。由此推断出抗逆转录病毒疗法 (ART) 是降低结核病发病率的原因之一。据估计,抗逆转录病毒疗法 (ART) 预防了 188 万(95% 置信区间,CI:1.65 至 2.11)艾滋病毒携带者患有结核病,或 15.7%(95% 置信区间:13.8 至 17.6)的 1196 万例艾滋病毒阳性结核病病例。异烟肼预防性疗法的人口覆盖率过低(合格者平均只有 1.0%)，无法对降低结核病发病率产生较大影响,结核病检测和治疗的改善与结核病发病率的降低呈弱相关性或无显著相关性。

**结论:**

抗逆转录病毒疗法 (ART) 与这 12 个国家结核病发病率的降低有关。因此，抗逆转录病毒疗法 (ART) 仍应是结核病-艾滋病毒合并感染高发病率地区的结核病防控重点，在治疗结核病方面仍需再接再厉。

## Резюме

**Цель:**

Исследование рекомендованных Всемирной организацией здравоохранения методов борьбы с туберкулезом, наиболее эффективно влияющих на снижение заболеваемости туберкулезом в 12 странах Африканского региона ВОЗ с высоким бременем туберкулеза, связанного с инфицированием вирусом иммунодефицита человека (ВИЧ).

**Методы:**

Авторы получили данные систем эпидемиологического надзора, исследований и лечения ВИЧ и туберкулеза за период с 2003 по 2016 год. Авторы использовали статистические модели для изучения эффективности антиретровирусной терапии и профилактического лечения изониазидом для снижения заболеваемости туберкулезом среди пациентов, страдающих ВИЧ. Также была исследована роль выявления и лечения случаев заболевания туберкулезом для предотвращения передачи *Mycobacterium tuberculosis* и, следовательно, для снижения заболеваемости туберкулезом.

**Результаты:**

В период с 2003 по 2016 год предоставление антиретровирусной терапии было связано со снижением заболеваемости туберкулезом в каждой из стран, а также с различиями в снижении заболеваемости туберкулезом между странами. Если исходить из того, что антиретровирусная терапия служила причиной снижения заболеваемости туберкулезом, она помогла предотвратить 1,88 млн случаев туберкулеза (95%-й ДИ: от 1,65 до 2,11) у пациентов, страдающих ВИЧ, или 15,7% (95%-й ДИ: от 13,8 до 17,6) из 11,96 млн ожидаемых случаев туберкулеза с сопутствующей инфекцией ВИЧ. Охват населения профилактическим лечением изониазидом был слишком низким (в среднем 1,0% лиц, отвечающих критериям), чтобы оказать существенное влияние на снижение заболеваемости туберкулезом, а улучшения в выявлении и лечении случаев туберкулеза были слабо или незначительно связаны со снижением заболеваемости туберкулезом.

**Вывод:**

Антиретровирусная терапия ассоциируется со снижением заболеваемости туберкулезом в исследованных 12 странах. Антиретровирусная терапия должна и впредь занимать центральное место в борьбе с туберкулезом в странах, где показатели туберкулеза с сопутствующей инфекцией ВИЧ высоки, но необходимо возобновить усилия в отношении лечения туберкулеза.

## Introduction

Among people with latent *Mycobacterium tuberculosis *infection, coinfection with the human immunodeficiency virus (HIV) carries a 10- to 15-fold risk of developing active tuberculosis.^1^^–^^3^ From 2003 to 2016, the countries worst-affected by the HIV epidemic, including Botswana, Eswatini and Lesotho reported national adult (15–49 years) HIV prevalence of over 20% and exceptionally high national tuberculosis incidence rates above 500 per 100 000 population per year (Table 1).^6^ Across the WHO African Region in 2016, there were an estimated 764 000 new cases of tuberculosis among people living with HIV, leading to 320 000 deaths.^6^

**Table 1:** Tuberculosis and HIV epidemics in 12 countries in the WHO African Region, 2003–2016

Country	HIV prevalence, %		Tuberculosis incidence per 100 000 people per year^c^		Change in tuberculosis incidence, % per year^d^		
	Total population^a^	Tuberculosis patients^b^	Total tuberculosis patients	HIV-positive tuberculosis patients	Total tuberculosis patients	HIV-positive tuberculosis patients	HIV-negative tuberculosis patients
Botswana	24	70	816	571	−6.9	−8.1	−4.0
Eswatini	27	84	1280	973	−5.8	−6.8	−2.2
Kenya	8	51	397	331	−3.8	−7.6	−1.2
Lesotho	24	77	1280	979	−3.7	−4.1	−2.1
Malawi	13	71	397	283	−5.9	−7.5	−3.1
Namibia	14	59	935	552	−5.7	−9.0	−2.5
Rwanda	4	45	102	46	−4.7	−5.2	−3.1
South Africa	17	65	977	607	−0.4	−0.5	−0.1
Uganda	8	59	248	146	−1.7	−4.3	1.0
United Republic of Tanzania	7	47	510	243	−4.4	−7.1	−2.7
Zambia	14	70	662	445	−4.3	−5.3	−2.7
Zimbabwe	19	78	617	471	−7.8	−8.7	−6.0

HIV: human immunodeficiency virus.

^a^ Prevalence of HIV infection in people aged 15–49 years.^4^

^b^ Values are maximum percentages of HIV infection in new tuberculosis patients.^5^

^c^ Values are maximum estimates of tuberculosis incidence and HIV-positive tuberculosis incidence.^5^

^d^ Average annual rate of change in HIV-positive, HIV-negative, and all tuberculosis incidence rate per 100 000 population. Negative values represent declining incidence.

To reduce the incidence of tuberculosis in populations with a high proportion of *M. tuberculosis* and HIV coinfection, the World Health Organization (WHO) and the Joint United Nations Programme on HIV/AIDS (UNAIDS) primarily recommend drug treatments such as isoniazid preventive therapy and antiretroviral therapy (ART) to prevent progression from latent to active tuberculosis. ART protects against tuberculosis by facilitating the restoration of immune function in people living with HIV, thus impeding progression of latent *M. tuberculosis* infection to active tuberculosis. Isoniazid preventive therapy, when used continuously with ART, generally gives coinfected adults additional protection from tuberculosis.^7^^–^^10^ WHO and UNAIDS also recommend combinations of drugs to prevent onward transmission of infection from patients with active tuberculosis.^6^^,^^11^ Combination chemotherapy and ART are the two principal interventions used to reduce tuberculosis incidence and have the greatest population coverage and longest history of use. The efficacy of all these treatments has been demonstrated in experimental and observational studies^7^^–^^9^^,^^12^^–^^14^ and drug treatments have the potential markedly to reduce incidence if high rates of treatment coverage can be achieved in target populations.^1^^,^^2^^,^^15^^–^^17^

The purpose of this study was to investigate which of these recommended drug treatment methods have had the greatest impact on tuberculosis incidence in 12 countries in the WHO African Region with high burdens of tuberculosis linked to HIV infection (further information available from our data repository).^18^

We used biologically-informed statistical models, together with data routinely collected annually since 1990, to examine the effects of ART and isoniazid preventive therapy in preventing tuberculosis disease in people living with HIV, and to investigate tuberculosis case detection and combination chemotherapy treatment as a means of interrupting *M. tuberculosis* transmission to reduce tuberculosis incidence. We used a retrospective longitudinal design to investigate each country and cross-sectional analysis to compare the countries.

## Methods

### Data sources

We obtained publicly available epidemiological surveillance, survey and treatment data on tuberculosis and HIV from WHO^5^ and UNAIDS.^4^ Population data are from the 2017 Revision of World Population Prospects.^19^

### Study setting

We chose 12 countries in the WHO African Region that had high tuberculosis–HIV coinfection rates and sufficient data to do this investigation: Botswana, Eswatini, Kenya, Lesotho, Malawi, Namibia, Rwanda, South Africa, Uganda, United Republic of Tanzania, Zambia and Zimbabwe. We excluded Mozambique because the trend in tuberculosis incidence was unclear from the series of annual case notifications: the number of cases reported increased continuously between 2002 and 2016, probably because the proportion of incident cases detected was increasing over that period and not because of a true rise in incidence (available from the data repository).^18^

### Intervention models

Since interventions to stop tuberculosis progression and transmission have different modes of action, we investigated the interventions, using two separate univariate models, rather than combining the interventions in one multivariate model.

We modelled the effect of ART on tuberculosis incidence by using the equation
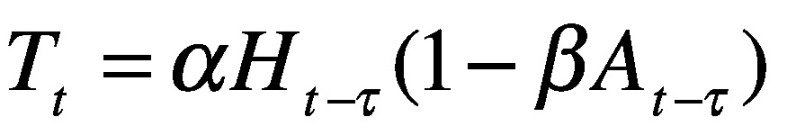
(1)where *T* is the annual incidence rate of tuberculosis among people living with HIV in year *t*. In this model *α* is the per capita incidence rate of tuberculosis in the untreated population of people living with HIV (*H*) after a delay of *τ* years, which depends on the prevalence and incubation period of *M. tuberculosis* in HIV-positive individuals and on the average age of HIV infection in the population (further details in the data repository).^18^ In the absence of drug treatment for either *M. tuberculosis* or HIV infection, *α* would change little over the period 2003–2016, so we took *α* to be constant over the time span of this study. *A* is the fraction of people living with HIV receiving ART, which takes effect after *τ* years, and coefficient *β* scales the effect of ART under the hypothesis that ART prevents tuberculosis. Neither this model, nor this analysis, accounts for the separate effects of ART on HIV, which are to interrupt viral transmission and increase population prevalence by improving survival and life expectancy.^20^^–^^22^

For each of the 12 countries, *T* is obtained from routine surveillance data (annual tuberculosis case notifications) and the estimated proportion of tuberculosis cases infected with HIV.^5^ Variables *H* and *A* are national estimates derived from national and subnational survey data.^4^ Rewriting the model as
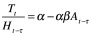
(2)parameters *α* and *β* are estimated from a linear regression of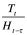 on *A_t-τ_*, with intercept *α* and gradient - *αβ*. The time delay, *τ*, was set at 2 years, based on the fit of the regression model to data, and on the recovery rate of CD4+ T-lymphocyte count after patients are started on ART (further details in the data repository).^18^^,^^23^ The number of people living with HIV that are prevented from developing tuberculosis is obtained from the fit of the model to data comparing the number of tuberculosis cases expected under the hypothesis that ART prevents (*β*  > 0, estimated) or does not prevent (*β*  = 0, counterfactual) tuberculosis.

**Fig. 1:**
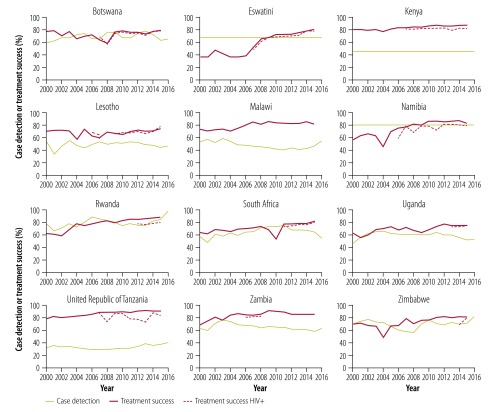
Trends in detection and treatment success of people with tuberculosis, 12 countries in the WHO African Region, 2000–2016

We modelled the effect of tuberculosis case detection and combination chemotherapy treatment on tuberculosis incidence, via reduced transmission, using the model
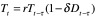
(3)where *T_t_*and *T_t-τ_* represent the incidence of all tuberculosis cases (regardless of HIV status) in the population. Coefficient *r* is the tuberculosis case reproduction number over the period *τ* in the absence of drug treatment. *D_t-τ_*is the proportion of all tuberculosis cases detected and successfully treated (that is, cases that converted from bacteriologically positive to negative, or completed treatment) and *δ* scales the effect of *D_t-τ_* in reducing *M. tuberculosis* transmission. Rewriting the model as
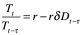
(4)parameters *r* and *δ* are estimated from a linear regression of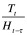 on *D_t-τ_*, with intercept *r* and gradient -*rδ*. The regression analysis is a test of whether an increase in case detection and treatment success accelerates the decline in tuberculosis incidence by reducing transmission. *D* is calculated from estimates of the proportion of all tuberculosis cases detected in any given year multiplied by the proportion successfully treated, which is reported by WHO in the following year (Fig. 1).^5^ The time delay, *τ*, was set at 2 years, but the conclusions of the analysis were not sensitive to the length of the delay (available from the data repository).^18^

HIV: human immunodeficiency virus.

Notes: Data are the estimated percentage of tuberculosis cases detected (constant through time in some countries), the measured proportion of tuberculosis cases successfully treated by combination chemotherapy, and the proportion of HIV-positive (HIV+) tuberculosis cases successfully treated where data are available.

We performed all analysis in Excel (Microsoft, Redmond, United States of America).

## Results

**Fig. 2:**
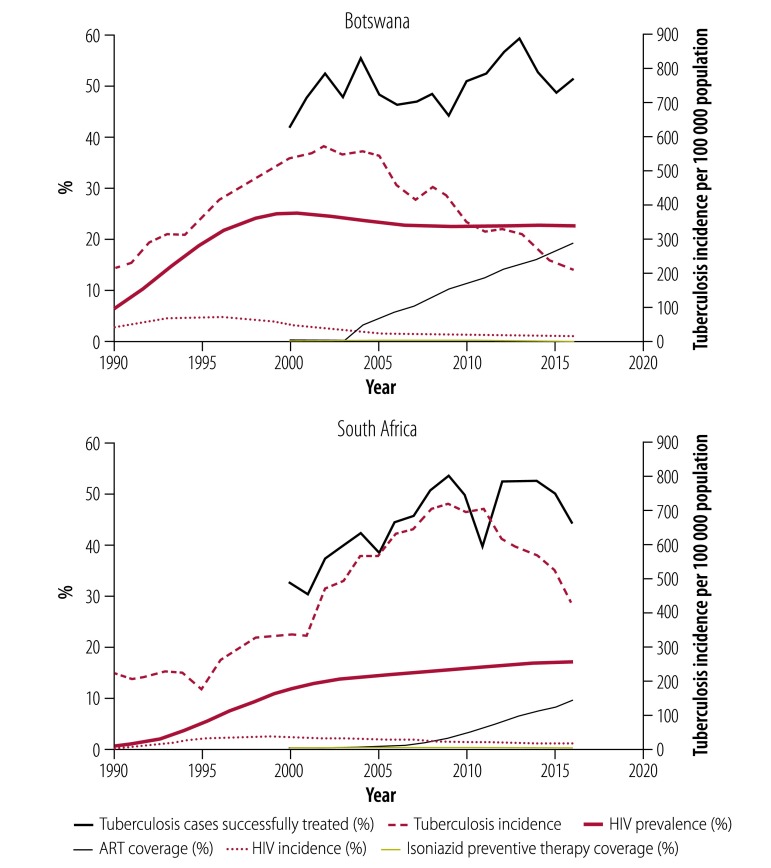
Trends in tuberculosis and HIV epidemics and treatments in Botswana and South Africa, 1990–2016

ART: antiretroviral therapy; HIV: human immunodeficiency virus.

Notes: Estimated HIV incidence and prevalence show data for adults (15–49 years). Coverages for ART and isoniazid preventive therapy are in the adult population.

Between 1990 and 2016, all 12 countries showed similar trends for tuberculosis and HIV epidemics and their control measures (available from the data repository).^18^ Fig. 2 shows Botswana and South Africa as examples. The numbers of tuberculosis cases reported each year increased following the rise of HIV incidence and prevalence. The subsequent downturn in tuberculosis incidence in both countries appeared to be associated with improvements in the proportion of tuberculosis cases successfully treated (that is, the proportion of cases detected × proportion successfully treated), but more obviously corresponded with the expanded coverage of ART. The scale-up of ART coverage from 2003 onwards was more rapid in Botswana than in South Africa, and the decline in tuberculosis incidence occurred earlier and more quickly. In the countries investigated here, the proportional decline and the absolute magnitude of the decline were greater for HIV-positive than for HIV-negative patients with tuberculosis (Table 1). The provision of isoniazid preventive therapy was low in both Botswana and South Africa, compared to the coverage of ART and to the proportion of tuberculosis patients treated with combination chemotherapy.

Data source: World Health Organization and the Joint United Nations Programme on HIV/AIDS.^4^^,^^5^

**Fig. 3:**
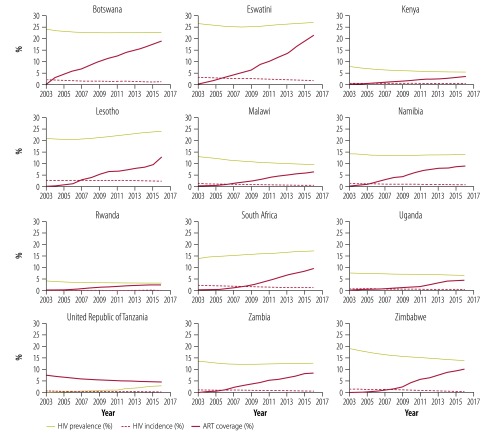
Trends in HIV incidence, HIV prevalence and ART coverage, 12 countries in the WHO African Region, 2003–2016

ART: antiretroviral therapy; HIV: human immunodeficiency virus.

Notes: The ratio of ART coverage to HIV prevalence is the coverage of ART in people living with HIV.

**Fig. 4:**
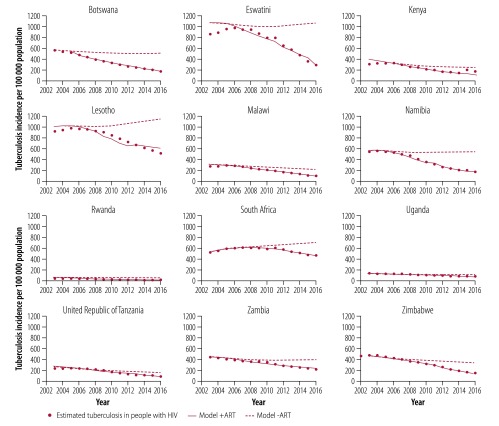
Trends in tuberculosis incidence in people living with HIV, 12 countries in the WHO African Region, 2003–2016

ART: antiretroviral therapy; HIV: human immunodeficiency virus.

Notes: Data are the estimated incidence of HIV-positive tuberculosis cases per 100 000 population per year. Solid lines represent the fit of the model under the hypothesis that ART prevents (for regression coefficients *β* > 1, lower lines) or does not prevent tuberculosis (counterfactual *β* = 0, upper lines). The number of cases prevented in each country is calculated from the area between upper and lower lines.

**Fig. 5:**
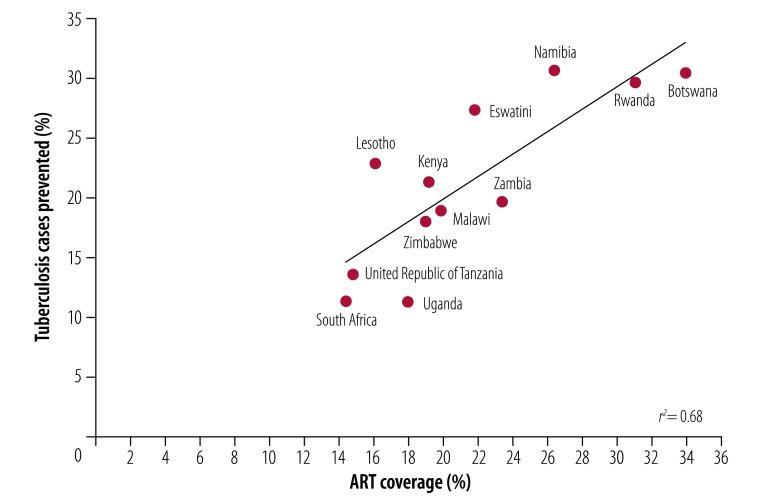
Association between antiretroviral coverage and tuberculosis incidence in people living with HIV, 12 countries in the WHO African Region, 2003–2016

ART: antiretroviral therapy; HIV: human immunodeficiency virus.

Notes: In our model, ART coverage explains 68% of the variation in the estimated percentage of HIV-positive tuberculosis cases prevented (*P* = 0.0009).

ART coverage increased in all 12 countries between 2003 and 2016 (Fig. 3) as tuberculosis incidence declined among people living with HIV (Fig. 4; data repository).^18^ While the changes in ART coverage and tuberculosis incidence are inversely correlated (available from the data repository),^18^ these correlations do not prove that ART was the cause of tuberculosis decline in these countries. Other unmeasured factors, including other interventions, could confound the correlations. However, the evidence for a causal role of ART is strengthened by comparing the results of regression analysis across all 12 countries: we found that the estimated fraction of HIV-positive tuberculosis cases prevented by ART was associated with the provision of ART across all countries (Fig. 5; *r*^2^ = 0.68; *P* < 0.001).

The average ART coverage was highest and contributed to the greatest proportions of HIV-positive tuberculosis cases prevented in Botswana, Namibia and Rwanda (Table 2). South Africa, the country with the largest number of people living with HIV (7.1 million in 2016),^11^ prevented the greatest number of HIV-positive tuberculosis cases (543 000) between 2003 and 2016, but the smallest fraction of such cases (11.2%; 542911/4846209) because ART coverage was low, on average 21.8%.

**Table 2:** Interventions to reduce tuberculosis incidence in 12 countries in the WHO African Region, 2003–2016

Country	Average ART coverage	Detection and treatment success of people with tuberculosis^a^		Average % of HIV-positive people on isoniazid preventive therapy^b^	No. of coinfected tuberculosis cases prevented (%)^c^
		Average(%)	Change(%/year)		
Botswana	46	51	12.5	0.51	48 000 (30)
Eswatini	33	39	91.9	0.17	54 000 (27)
Kenya	28	38	6.3	0.56	392 000 (21)
Lesotho	23	33	10.4	0.11	75 000 (23)
Malawi	29	51	−9.0	3.09	111 000 (19)
Namibia	36	61	30.5	0.44	55 000 (31)
Rwanda	42	62	14.8	0.01	19 000 (30)
South Africa	22	47	28.2	4.21	543 000 (11)
Uganda	27	44	−0.4	0.001	72 000 (11)
United Republic of Tanzania	23	28	16.9	0.18	195 000 (13)
Zambia	33	56	−7.5	0.02	165 000 (20)
Zimbabwe	29	49	21.1	2.93	149 000 (18)

ART: antiretroviral therapy; HIV: human immunodeficiency virus.

^a^ Average and change in the number of tuberculosis cases detected and successfully treated for the years 2003 to 2016.^5^

^b^ Coverage (%) calculated as: total number of people living with HIV who start treatment/total number of people living with HIV, aged 15–49 years.

^c^ Estimated number and percentage of HIV-positive tuberculosis cases prevented by ART, 2003–2016.

Based on the fit of the model to data for each of the countries between 2003 and 2016 (Fig. 4; data repository),^18^ ART prevented 1.88 million (95% confidence interval, CI: 1.65 to 2.11) people living with HIV from developing active tuberculosis, or 15.7% (95% CI: 13.8 to 17.6) of the 11.96 million HIV-positive tuberculosis cases expected. An alternative way of calculating the number of cases prevented is to use the association between cases prevented and ART coverage across the 12 countries (Fig. 5): summing the predictions for each country gives a similar total of 2.06 million people living with HIV prevented from developing active tuberculosis over the same period, or 17.3% of the number of HIV-positive cases expected.

Although the primary effect of ART is to stop progression from *M. tuberculosis* infection to tuberculosis disease in people living with HIV, the consequent reduction in tuberculosis incidence might also reduce onward transmission. The incidence of tuberculosis in HIV-negative people declined in all countries alongside tuberculosis in HIV-positive people, albeit more slowly (Table 1) and, comparing countries, the rates of decline in HIV-positive and HIV-negative tuberculosis were correlated (*r*^2^ = 0.48; *P* = 0.02).

Considering the treatment of tuberculosis infection or disease (rather than of HIV infection), neither combination chemotherapy for tuberculosis, nor the prophylactic treatment of *M. tuberculosis* infection with isoniazid, markedly accelerated the decline in tuberculosis incidence between 2003 and 2016. The evidence is as follows: First, the detection of infectious pulmonary tuberculosis cases at an early stage of illness followed by successful drug treatment should interrupt the transmission of *M. tuberculosis* from all patients regardless of HIV status, thereby reducing tuberculosis incidence in the rest of the population.^3^^,^^15^^,^^17^ Comparing countries, the rate of decline in tuberculosis incidence is expected to be faster where case detection and treatment success are higher on average. The national proportions of all tuberculosis cases detected and successfully treated varied between 27.5% and 62.2% (Table 2). There was no discernible association between tuberculosis case detection or treatment and tuberculosis incidence across the countries (Table 2; *r*^2^ = 0.06, P = 0.65).

If case detection and treatment success improve with time in any individual country, we expect to see an accelerated decline in tuberculosis incidence as the interruption of transmission lowers the tuberculosis case reproduction number. Tuberculosis case detection and treatment did improve somewhat in most countries between 2003 and 2016 as tuberculosis incidence declined, though not in Malawi, Uganda and Zambia (Table 2; Fig. 1). Within each country, there were either weak associations (Eswatini, South Africa, Zimbabwe) or no significant associations (the other nine countries) between tuberculosis case detection and treatment success, and the rate of decline in tuberculosis incidence (available from the data repository).^18^ In summary, neither the levels nor the change in tuberculosis case detection and treatment success can explain the reduction in tuberculosis incidence observed in all 12 countries from around 2003 onwards (Fig. 4).

Second, we examined isoniazid preventive therapy in the 12 countries and found that the coverage of such therapy among people living with HIV was generally low between the years 2003 and 2016 (average 1.0% of persons eligible across countries; Table 2). In 2016, South Africa started the largest number of people on isoniazid preventive therapy, 385 932 people, or 51.3% of those 751620 people newly enrolled in care for HIV/acquired immunodeficiency syndrome (AIDS) that year. However, only 4.2% of people living with HIV, on average, received isoniazid preventive therapy between 2003 and 2016, contrasted with an average of 21.8% who started ART during the same period. In comparison, an average 3.1% of people living with HIV in Malawi and 2.9% of people living with HIV in Zimbabwe received isoniazid preventive therapy. These three countries together reported 91.0% (3.28 million/3.61 million) of all people living with HIV who had started isoniazid preventive therapy in the 12 countries by 2016. Thus, isoniazid preventive therapy may have protected thousands of coinfected people from developing tuberculosis in Malawi, South Africa and Zimbabwe, but the effect on tuberculosis incidence at a population level would have been small in these three countries, and negligible in the other nine countries.

## Discussion

Our analysis suggests that the roll-out of ART, supported by the services needed to provide treatment, such as HIV testing, drug procurement, treatment supervision, outcome monitoring and nutritional supplements, has played a major part in the tuberculosis decline in southern and eastern Africa since 2003. Our results are broadly in line with a previous study^24^ that also found an association between ART coverage and the reduction in tuberculosis incidence among people living with HIV.

By stopping the progression from *M. tuberculosis* infection to tuberculosis disease, we estimated that ART prevented 1.88 million HIV-positive tuberculosis cases between 2003 and 2016. If ART also reduced the transmission of *M. tuberculosis* infections, as suggested by this analysis, then our estimate of prevented tuberculosis cases would be conservative. In contrast, we did not identify any evidence that drug treatment of tuberculosis infection or disease played more than a minor role in accelerating the rate of tuberculosis decline.

This study has limitations. Given the well-known uncertainties in measuring the coverage of tuberculosis control programmes, we cannot be sure that improvements in case detection and treatment success did not accelerate the decline in tuberculosis incidence. Moreover, our study was an analysis of change; we did not investigate, and therefore cannot quantify, the effect of a constant rate of case detection and treatment in reducing transmission and tuberculosis incidence.

We found large variations among countries in the estimated fraction of tuberculosis cases prevented by ART. These variations were linked to differences in ART coverage. Future research should investigate both the immediate and deeper reasons for differences in coverage, because these reasons are likely to influence the future success of HIV and tuberculosis control programmes. Between 2003 and 2016, Botswana prevented the largest fraction of tuberculosis cases and achieved the highest coverage of ART. Since independence in 1966, Botswana has invested in public services to tackle ill health and poverty^25^ and was the first African country to provide universal, free-of-charge ART.^26^ South Africa prevented the smallest fraction of tuberculosis cases among the countries investigated. Following controversy over the link between HIV and AIDS,^27^ South Africa was slower to expand treatment for people living with HIV, but now has the largest number of people on ART and isoniazid preventive therapy worldwide.^6^^,^^11^ In 2015, South Africa became the first country in sub-Saharan Africa to issue full regulatory approval for pre-exposure prophylaxis, using ART to protect HIV-negative people from acquiring HIV infection.^28^ Consistent with these recent developments in South Africa, the rate of tuberculosis decline among people living with HIV increased to 4.6% per year between 2010 and 2016 (compared with the average decline of 0.5% per year over the period 2003–2016; Table 1).

The number of patients with tuberculosis tested for HIV infection across Africa has greatly increased. In 2016, 82% of reported tuberculosis patients had a documented HIV test result, up from 2% in 2004.^6^ Once tested positive for HIV, tuberculosis patients become eligible for ART, increasing the chance of successful tuberculosis treatment and reducing the risk of death. However, far less progress has been made in finding and treating tuberculosis among people living with HIV and among HIV-negative people living in settings with high prevalence of coinfection, both forms of progress are imperative for control, and ultimately, for the elimination of tuberculosis.^3^ To find individuals with infectious tuberculosis earlier requires a greater awareness of the risk of tuberculosis among people living with HIV and a more active approach to tuberculosis diagnosis at home, in the community and in health facilities.^29^^–^^32^

While millions of people living with HIV now receive ART continuously in southern and eastern Africa, a small number also receive isoniazid preventive therapy.^6^^,^^10^ In this context, the success of Malawi, South Africa and Zimbabwe in starting patients on isoniazid preventive therapy will improve knowledge on how to identify candidates for isoniazid preventive therapy (recognizing that efficacy is greater for those who are tuberculin skin test positive and that patients with tuberculosis disease must be excluded), maintain adherence to prolonged prophylaxis (daily treatment for at least 6 months), and manage the adverse reactions that affect a small fraction of patients on isoniazid.^33^

Tuberculosis accounted for 1.7 million deaths in 2016, including those among HIV-positive cases^6^ and tuberculosis incidence is declining too slowly to reach international targets. For example, the WHO End TB Strategy aims to cut the tuberculosis incidence worldwide by 80% between 2015 and 2030, which requires a fivefold increase in the rate of decline, from 2% per year at present to at least 10% per year.

The slow pace of technological development in tuberculosis control, including diagnostics, drugs and vaccines^6^, provides a strong argument for making the best use of the tools that are currently available. The best strategies may vary from one setting to another, but for populations having a high or increasing prevalence of tuberculosis–HIV coinfection, ART is an effective intervention. However, the scale-up of ART alone will not be enough to reach international targets. Besides the search for new interventions and technologies, additional efforts are needed to maximize the benefits of directly treating tuberculosis infection and disease.
